# The incidence of paediatric ACL injury is increasing in Finland

**DOI:** 10.1007/s00167-019-05553-9

**Published:** 2019-06-20

**Authors:** Frederick K. Weitz, Petri J. Sillanpää, Ville M. Mattila

**Affiliations:** 1 Department of Paediatric Surgery, University of Tampere, Teiskontie 35, 33521 Tampere, Finland; 2 Pihlajalinna Koskisairaala Hospital, Hatanpään Valtatie 1, 33100 Tampere, Finland; 3 Department of Orthopaedic Surgery, University of Tampere, Teiskontie 35, 33521 Tampere, Finland

**Keywords:** Paediatric ACL rupture, Adolescent ACL rupture

## Abstract

**Purpose:**

Anterior cruciate ligament (ACL) injury is a common knee injury in paediatric and adolescent patients. The population-based incidence of paediatric ACL injury is, however, unknown. Recent studies suggest increased ACL injury rates among adolescents, especially in active, sports-participating population. The purpose of this study was to investigate the population-based incidence rates of ACL injuries and trends in paediatric ACL reconstruction surgery.

**Method:**

All ACL injuries were identified (ICD-10 diagnosis code S83.5) leading to hospitalisation or surgery using validated Finnish National Hospital Discharge Register (NHDR) data from 1997 to 2014. The sample comprised 19,961,205 Finnish residents aged less than 18 years at the time of injury. Hospital admissions with the diagnosis code S83.5 were analysed more thoroughly including, sex, age and the need for surgical interventions.

**Results:**

During the 18-year study period, 4725 subjects of the study population had sustained an ACL injury. The total ACL injury incidence in study population was 23.3 per 100,000 person-years. The median age of the patients at the time of injury was 16 years (range 4–17). The incidence of ACL injury increased with age, and the highest incidence was observed among 17-year old (113.5 per 100,000 person-years). Incidence rate did not differ between genders. From the total ACL injury population of 4725 hospitalisations, 3168 (67.0%) underwent ACL reconstruction, of which 2988 (94.3%) were treated with arthroscopic reconstruction and 180 (5.6%) with open surgery. In addition, 1557 (33.0%) were treated non-operatively without ACL reconstruction. The annual incidence of ACL injuries in the Finnish paediatric population has increased during the past 15 years. The lowest incidence rate was seen in 1999 (incidence of 17.7 per 100,000 person-years, 195 ACL injuries) and the highest in 2011 (incidence of 31.5 per 100,000 person-years, 346 ACL injuries). The highest increase in ACL injuries was seen in girls aged 13–15 years, with an increase of 143%.

**Conclusion:**

ACL injury is not a negligible knee injury in the paediatric population. The incidence of paediatric ACL injury has increased during the past 15 years. Moreover, a twofold increase in incidence of paediatric ACL injury was noted during the last 10 years of the study period. Incidence rates among male and female paediatric patients were comparable.

**Level of evidence:**

III.

## Introduction

ACL injury is the most common knee injury in physically active adolescents. The true population-based incidence of ACL injury in adolescents is, however, unknown [1]. The findings of a previous cohort study from Finland showed that the incidence of ACL injury was 60.9 per 100,000 person-years among median 22-year old adults [2]. Furthermore, some recent reports have suggested that the incidence of paediatric and adolescent ACL injury is increasing [3–5]. Although true nationwide incidence rates remain unknown, various studies have reported high rates of ACL injuries in selected skeletally immature populations [6–8].

The highest individual risk for ACL injury has been reported among 15–24-year-old female athletes who participate in sports that require sudden deceleration, landing and pivoting movements [9–11]. A recent study [12] using data from the national database of the United States reported a significant increase in both paediatric and adolescent ACL injuries as well as in ACL reconstructions. Interestingly, the increase in ACL injury rates in the child and adolescent populations seems to be significantly higher than the increase seen in the adult population [12].

The treatment of paediatric ACL injuries has remained controversial mainly due to the demanding surgical techniques involved and the potential risk for surgical complications, such as growth disturbance. To date, various surgical techniques for paediatric ACL reconstruction have been described in the literature. In some patients, nonoperative treatment has been shown to be an option [13–15], but there have been some studies that have reported poor outcomes in paediatric and adolescent patients non-operatively treated for ACL injury [16]. Aichroth et al. [17] attempted to manage ACL injured patients with open physes by rehabilitation, modification of physical activity and bracing. At 72-month follow-up post ACL injury, however, the nonoperative treatment resulted in a significant decrease in Lysholm score and signs of early osteoarthritis [17].

Since the incidence of adolescent ACL injury is unknown, the aim of this present study was to assess the population-based incidence and trends in paediatric and adolescent ACL injury leading to hospitalisation or surgery in Finland between 1997 and 2014. The sample comprised the nationwide ACL injury population aged less than 18 years at the time of injury.

## Materials and methods

The nationwide data from the Finnish National Hospital Discharge Register (NHDR) was reviewed for the time period from 1997 to 2014. Founded in 1967, the NHDR includes all Finnish residents and contains data on age, sex, domicile, type of hospital (public or private), length of hospital stay, primary and secondary diagnosis and operations performed during the hospital stay. The study covered the whole paediatric and adolescent population averaging an annual 1.1 million persons (aged < 18 years) in Finland during the 18-year study period from January 1, 1997 to December 31, 2014. The study was approved in the Ethics Committee (Dnro THL/82/5.05.00/2011). For incidence of ACL injury, we divided the population into three subgroups according to age: (1) 0–12 years (2) 13–15 years and (3) 16–17 years and calculated the incidence in these groups. Furthermore, male and female patients were analysed separately. The inclusion criteria for this study were hospitalisation and surgery with primary or secondary ICD-10 code S83.5. The Nomesco Classification of Surgical Procedures (Finnish version) treatment codes NGE35 and NGE30 were used for arthroscopic and open ACL reconstruction.

### Statistical analysis

The primary outcome variable of this study was the incidence of ACL injury per 100,000 person-years in patients aged less than 18 years. The incidence rates were calculated using the annual adolescent population size (averaging 1.1 million per/year during the study period) obtained from the statistical database in Finland, a statutory electronic national population register [http://www.stat.fi/til/vaerak/index_en.html]. All Finnish citizens have a social security number that allows identification of the specific number of citizens each year and each hospitalisation occurring on the individuals’ social security number. The true incidence was based on the entire population of persons aged ≤ 18 years in Finland, and thus avoided the bias in hospital region or cohort-based estimates. Accordingly, confidence intervals were not calculated. Statistical analyses were performed using SPSS 23.0 software.

## Results

During the 18-year study period, 4725 subjects of the study population had sustained an ACL injury. The total ACL injury incidence in our population was 23.3 per 100,000 person-years. The median age of the patients was 16 years (range 4–17), and the median hospitalisation time was one day in all the groups (range from 1 to 28 days).

**Fig. 1:**
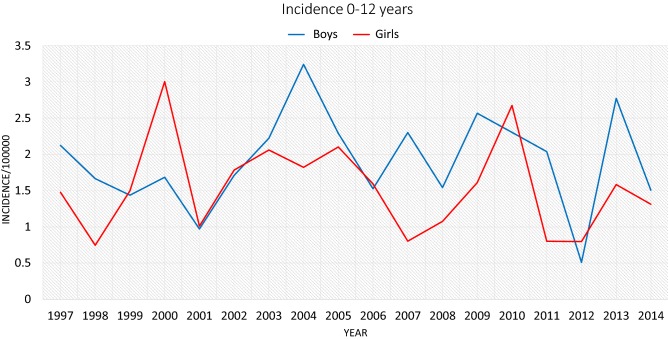
The incidence of ACL rupture in children aged 0–12

**Fig. 2:**
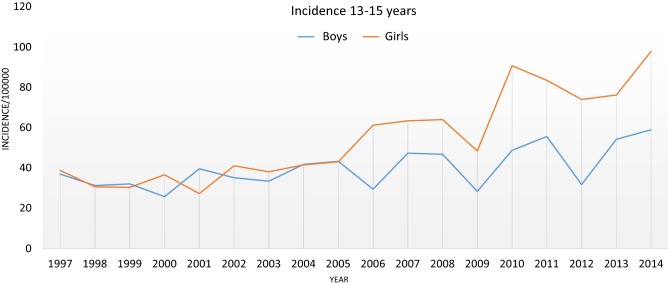
The incidence of ACL rupture in children aged 13–15

The incidence of ACL injury increased with age. The incidence of ACL injury in the age group 0–12 was the same for both genders varying from 0.7 to 3.0 per 100,000 person-years (Fig. 1) in girls and 0.5–3.2 per 100,000 person-years (Fig. 2) in boys. In this age group, there was no significant variation in the incidence over the 18-year study period.

**Fig. 3:**
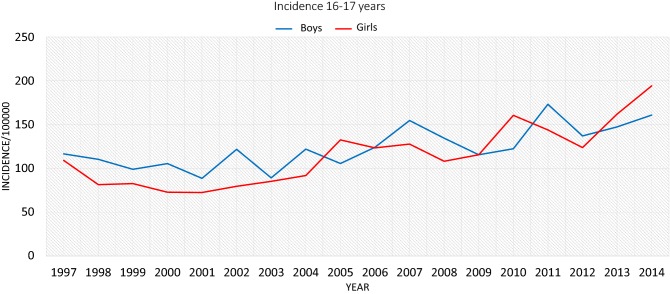
The incidence of ACL rupture in children aged 16–17

The incidence of ACL injury in the older age groups (13–15 and 16–17) increased from 1997 to 2014. During the early part of the study period from 1997 to 2000, the incidence of ACL injuries was on average 34.1 per 100,000 person-years in girls aged 13–15 and 86.3 per 100,000 person-years in girls aged 16–17 (Fig. 3). For males during the same period, the incidence rate was 31.5 per 100,000 person-years in boys aged 13–15 and 107.7 per 100,000 person-years in boys aged 16–17, respectively. Correspondingly, in girls aged 13–15 years, the incidence rates increased from 34.1 per 100,000 person-years in the late 1990s to 86.3 per 100,000 person-years in the early 2010s (2010–2014). The incidence of ACL injury in this group, therefore, increased by a remarkable 143%. The increase in girls aged 16–17 was 81%, when the incidence rates of the late 1990s are compared to those of the early 2010s. In boys aged 13–15, the increase in ACL injury was 59%, and in boys aged 16–17 the increase was 44%.

From the 4725 patients hospitalised with ACL injury, 2988 (63.2%) were treated with an arthroscopic ACL reconstruction; 180 (3.8%) were treated with open ACL reconstruction and 1557 (33%) underwent non-operative management for ACL injury. NHDR does not include data on details of different surgical techniques since arthroscopic ACL reconstruction is with the same code, and thus it is not possible to differ between different surgical approaches. This data does not give information over the surgical results and possible complications in form of re-rupture or growth disturbance.

## Discussion

The most important finding of the present study was the over twofold increase of ACL injuries in Finnish population in age group 13–17 years in both genders. During the 18-year study period, 4725 subjects of the study population had sustained an ACL injury. The total ACL injury incidence in our population was 23.3 per 100,000 person-years and the median age of the patients was 16 years (range from 4 to 17). The incidence of ACL injury is increasing in the paediatric and adolescent population. To the best of our knowledge, a population-based incidence of paediatric ACL injury has not been previously reported, although [1] recent studies have suggested increased ACL injury rates among adolescents participating in sports activities. The main finding in the present study was the remarkable high increase in the incidence of ACL injury in the adolescent population aged over 12 years in both sexes. The most significant increase in the ACL injury incidence was seen in girls aged 16–17 years. In children aged less than 12 years, the incidence of ACL injury remained constant during the 18-year study period. The highest increase (143%) in incidence of ACL injuries was seen in girls aged between 13 and 15 years. This finding is supported by some of the previous studies [18, 19]. For example, Werner et al. [12] reported a significant increase in paediatric and adolescent ACL tear diagnosis and reconstruction compared with adult patients. It is assumed that these increases of ACL rupture incidence are mostly due to increased participation in competitive sports, especially in girls. The availability of MRI examinations might have increased in Finland during the study period. In this study no access was given to the information of the total amount of MRI investigation done yearly.

In addition to incidence rate, we analysed the distribution of different treatment modalities for ACL injury. In our data from the 4725 hospitalised patients with ACL injury, 2988 (63.2%) were treated with arthroscopic ACL reconstruction, 180 (3.8%) were treated with open ACL reconstruction and 1557 (33.0%) had no ACL reconstructive surgery during the study period. The median age at the time of surgery was 16 years, indicating a tendency towards ACL reconstruction surgery for skeletally mature subjects and nonoperative management for skeletally immature subjects. In a study by Morgan et al. [20], the median age was similar to our study with 16 years and 6 months. The patients from the Morgan et al.’s study [20] were aged 13–18.

Due to the potential risk for the growth disturbances associated with ACL reconstruction surgery, the management of paediatric ACL injury has been controversial in skeletally immature subjects. In addition, current surgical methods require a high level of expertise in knee surgery that focuses on avoiding growth disturbances either through indirect growth changes in extraepiphyseal surgical procedures or through growth plate injuries in epiphyseal or transphyseal procedures [7, 21–24]. Two recent reviews have stated [25, 26] that paediatric ACL reconstruction is safe regardless of the technique used. The reported overall rate of growth disturbance was 2.6% after paediatric ACL reconstruction [26]. Regarding the timing of surgery, in their meta-analysis, Dunn [25] et al. favoured early operative treatment for paediatric patients with ACL tears over delayed or non-operative treatment. The non-operative group had a significant higher risk for a meniscal tear after primary injury, and in this group, 75% of the patient reported instability, whereas in the operative group only 13.6% reported instability of the knee. The authors recommend early operative treatment for paediatric patients to prevent instability related problems.

The strength of this study was the nationwide Finnish National Hospital Discharge Register (NHDR) database that comprises all the ACL injuries that lead to hospitalisation or surgical intervention. Therefore, using nationwide database analysis, the risk for selection bias in geographic region or hospital-based population analysis was avoided. In the previous studies, the accuracy of the Finnish National Discharge Register has been shown to be excellent [27, 28].

A limitation of the study was that the database may not include those patients with ACL injury who may have been treated as an outpatient without any kind of surgical interventions during the study period. Most likely these patients would have been close to 18 years of age at the time of injury. The interventions would, therefore, have been performed and registered after the patient reached 18 years of age, and thus would have led to exclusion from the less than 18-years old study population. Consequently, this study may have slightly underestimated the increase in incidence of ACL injuries in patients aged 17 or older. Another limitation is that the hospital discharge register data used in this study does not allow differentiation between ACL reconstructions performed on skeletally immature patients and mature patients. Whether the rate of ACL reconstructions in the skeletally immature population is increasing at the same level as the incidence of ACL injury, needs to be further investigated.

In this nationwide population-based study, it was found that the incidence of paediatric ACL injury increased during the 18-year study period. A surprisingly high, nearly twofold increase in incidence of paediatric ACL injury was noted during the last 10 years of the study period. The incidence rates among male and female paediatric patients were comparable. Efforts to prevent ACL injury should probably be more focused on the paediatric and adolescent populations in the near future due to the concerning increase in injury rates. Moreover, the long-term prognosis related to age and skeletal maturity at the time of ACL injury and the role of different treatment modalities warrant further research.

## Conclusion

This study shows a more than twofold increase of ACL injury incidence in adolescent population of Finland. Especially girls aged 13–17 had a significant increase of ACL injury incidence. Most of this increase can be explained with the increase of competitive sports participation and somewhat also with better imagining possibilities during the study period. To reduce the increasing occurrence of ACL injuries work has to be done in the athletic teams with preventive measures.
